# Intermittent vs continuous electrocardiogram event recording for detection of atrial fibrillation—Compliance and ease of use in an ambulatory elderly population

**DOI:** 10.1002/clc.23323

**Published:** 2020-01-09

**Authors:** Tove Fredriksson, Katrin Kemp Gudmundsdottir, Viveka Frykman, Leif Friberg, Faris Al‐Khalili, Johan Engdahl, Emma Svennberg

**Affiliations:** ^1^ Department of Clinical Sciences Danderyd University Hospital, Karolinska Institutet Stockholm Sweden

**Keywords:** ambulatory ECG, atrial fibrillation, electrocardiography, epidemiology

## Abstract

**Background:**

There are many atrial fibrillation (AF) screening devices available. Validation studies have mainly been performed in optimal settings in the young population.

**Hypothesis:**

We aim to compare the yield of AF detection, compliance, and patient‐based experience in an ambulatory elderly population by using intermittent electrocardiogram (ECG) recordings and continuous event recording simultaneously.

**Methods:**

The study participants were part of the STROKESTOP II study, a Swedish screening study for AF. All participants were 75/76 years of age, were clinically free of AF, and had N‐terminal pro b‐type natriuretic peptides levels ≥125 ng/L. AF screening was performed in parallel during a 2‐week period, using a continuous event recording device (R‐test 4; Novacor) and 30‐second intermittent recordings using a handheld ECG device (Zenicor II) four times daily. Participants were asked to fill out a questionnaire with regard to compliance and ease of use of the devices.

**Results:**

During continuous event recording, 6% (n = 15/269) were diagnosed with AF and intermittent ECG detected AF in 2% (n = 5/269) of the participants (*P* = .002). No new cases of AF were detected using intermittent ECG monitoring only, but some episodes were detected in parallel for patients. On a graded ordinal scale of 1 to 5, with 1 reflecting “very easy to use”, continuous monitoring was graded 2 (interquartile range [IQR]: 1‐3) compared to intermittent 1 (IQR: 1‐1) (*P* < .001).

**Conclusion:**

Continuous event recording detected three times more AF compared to intermittent ECG in an elderly ambulatory population. Compliance and user‐friendliness were rated higher for the intermittent ECG device.

## 1 BACKGROUND

Atrial fibrillation (AF) is the most common sustained cardiac arrhythmia. The prevalence of clinically diagnosed AF is approximately 3% in the adult population,^1^, ^2^, ^3^ but increases steeply with age.^1^, ^4^ At least 20% of all strokes are directly attributable to AF.^5^ The attributable risk of AF to stroke increases with higher age which is in contrast to other risk factors for stroke.^6^ Oral anticoagulant treatment in AF patients leads to a marked decrease in stroke risk.^7^

AF can be asymptomatic, and individuals with asymptomatic AF have been suggested to have a higher risk of stroke than those with symptomatic AF.^8^, ^9^ AF screening can facilitate early detection of AF. According to current European Society of Cardiology guidelines, opportunistic screening in populations aged >65 years is recommended, by pulse palpation or electrocardiogram (ECG) rhythm strip. Systematic screening may be considered in individuals aged >75 years or with high risk of stroke.^10^ Prolonged screening has been shown to detect six times more AF in an elderly population compared to single‐time point ECG.^11^

With the advent of new technology, many new devices for AF detection have been developed. The validation of these devices is commonly performed in an optimal in‐office setting, in the young population.^12^ However, arrhythmia affects mainly elderly and arrhythmia is seldom present at the time of hospital visit. Hence, there is a need to validate and compare methods for AF screening in an ambulatory setting in the intended population.^13^

We aim to compare the yield of AF detection, compliance, and patient‐based experience in population screening in elderly individuals, by using intermittent ECG recordings vs continuous event recording.

## 2 METHODS

### 2.1 Study population

This is a substudy of STROKESTOP II, a Swedish mass‐screening study for AF in individuals aged 75 and 76 years. The study protocol has been published previously.^14^ In short, all inhabitants born in 1940 and 1941 in Stockholm County (n = 28 712) were randomized to screening or to a control group with inclusion from April 2016 to March 2018. Participants in the screening group without a prior diagnosis of AF and N‐terminal pro b‐type natriuretic peptides levels (NT‐proBNP) ≥125 ng/L were asked to perform intermittent ECG recordings for 30 seconds four times daily for 2 weeks using a one‐lead ambulatory handheld Zenicor II device (Zenicor Medical Systems, Stockholm, Sweden).

### 2.2 Inclusion

Consecutive participants were included during the last 8 months of the STROKESTOP II study. All participants were free of AF at baseline and had NT‐proBNP ≥125 ng/L. All participants received oral and written information about the substudy and provided informed consent.

### 2.3 Screening procedure

During inclusion in the STROKESTOP II study, all participants filled out a health questionnaire from which baseline medical data were gathered. In addition to the one‐lead ambulatory handheld Zenicor II device used in STROKESTOP II, participants were equipped with a one‐lead continuous event recorder, R‐test 4 evolution (Novacor, Rueil Malmasion, France), and were instructed to use the recording devices in parallel for 2 weeks. Both devices had buttons for activation if symptomatic arrhythmia occurred. The participants were also asked to fill out a questionnaire with regards to their experience of the two different AF screening devices including information on completion of the 2‐week registration (yes/no), problems leading to discontinuation (free text), ease of use (on a graded ordinal scale 1‐5), and effect on daily life. They were also asked to fill out a symptom diary during the 2 weeks.

### 2.4 Intermittent ECG

To identify ECGs with suspected AF, all intermittent recordings were inspected manually in addition to the validated computerized algorithm used by Zenicor.^15^ The Zenicor device has been validated with 92% sensitivity and 96% specificity for AF detection compared to a 12‐lead ECG.^12^

### 2.5 Continuous event recording

The R‐test 4 evolution device was programmed to store not only AF suspicious activity, but also other significant arrhythmias (Table S1). The R‐test 4 has a monitoring capacity of 32 days and can store a total of 60‐minute ECG recording. We chose to interpret arrhythmia episodes automatically displayed by the system, as this reflects normal usage of the device. The device automatically displays the 42 most typical episodes of suspected AF and 10 episodes of each other arrhythmia category. In 15% of the participants, all accessible ECGs were analyzed, without additional arrhythmia diagnosed by extending the manual examination. The algorithm of the R‐test 4 device has been validated compared to continuous ECG and has 92% sensitivity and 87% specificity for AF detection.^16^

### 2.6 Diagnostic criteria for AF

The diagnostic criteria for AF used in the study are according to ESC guidelines: absolute irregular rate‐to‐rate intervals, no discernable, distinct *p*‐waves, and duration of at least 30 seconds.^10^ All participants diagnosed with AF were offered cardiologist follow‐up.

### 2.7 Other significant arrhythmias

Participants with other significant bradyarrhythmias such as second‐degree atrioventricular block Mobitz type II, sinoatrial block or sinoatrial arrest for >2 seconds during daytime or >3 seconds at night‐time, or sinus bradycardia with a frequency of less than 30 beats/min were offered cardiologist follow‐up. Similar follow‐up was offered to participants with multifocal or broad complex tachycardia consisting of eight or more consecutive beats.

### 2.8 Statistical methods

All continuous variables were analyzed according to non‐normal distribution, as most variables were non‐normally distributed. Together with all ordinal data, they were reported as median with interquartile range (IQR), and analyzed using Mann‐Whitney *U* test. Chi‐square test was used for proportions. Comparisons of the two screening methods were performed using McNemar's test for dichotomous variables and paired sample t‐test for continuous variables. All tests were two‐sided, and a value of *P* < .05 was regarded as significant. All analyses were performed using IBM SPSS Statistics, version 24 software (IBM Corp, Somers, New York).

### 2.9 Ethics

The study complies with the Declaration of Helsinki. The protocol was approved by the regional ethics committee (DNR 2015/2079‐31/1, 2016/852‐32, and 2017/527‐32). All participants provided informed consent.

## 3 RESULTS

Of the 3763 participants in STROKESTOP II, 269 (7%) were included in this comparison study between June 2017 and January 2018.

### 3.1 Newly diagnosed AF per screening method

**Figure 1:**
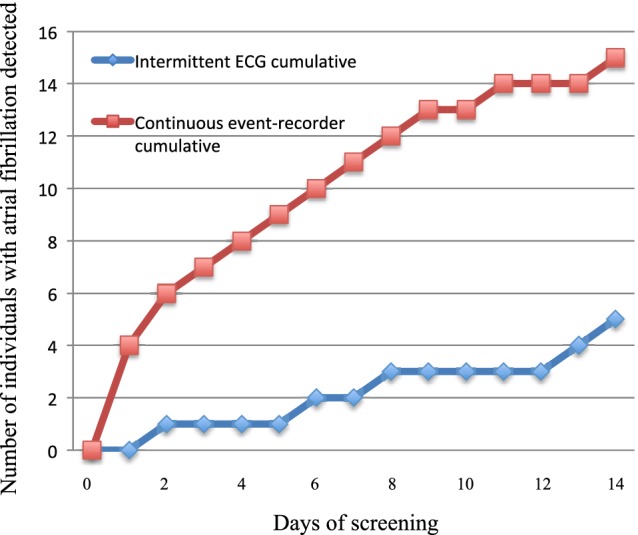
Time to first detection of atrial fibrillation per screening method (note that all participants are included in both groups)

Continuous event recording detected AF in 6% (n = 15) of the participants and intermittent ECG detected AF in 2% (n = 5) (*P* = .002). Using parallel monitoring, no new cases of AF were detected using intermittent ECG monitoring only. Using continuous event recording, AF was detected on average day 4 (IQR: 1‐8) compared to day 8 (IQR: 4‐14) using intermittent ECG (*P* = .135). There was a significant difference in AF detection between the two devices already after 3 days of monitoring (*P* = 0.03; Figure 1).

**Figure 2:**
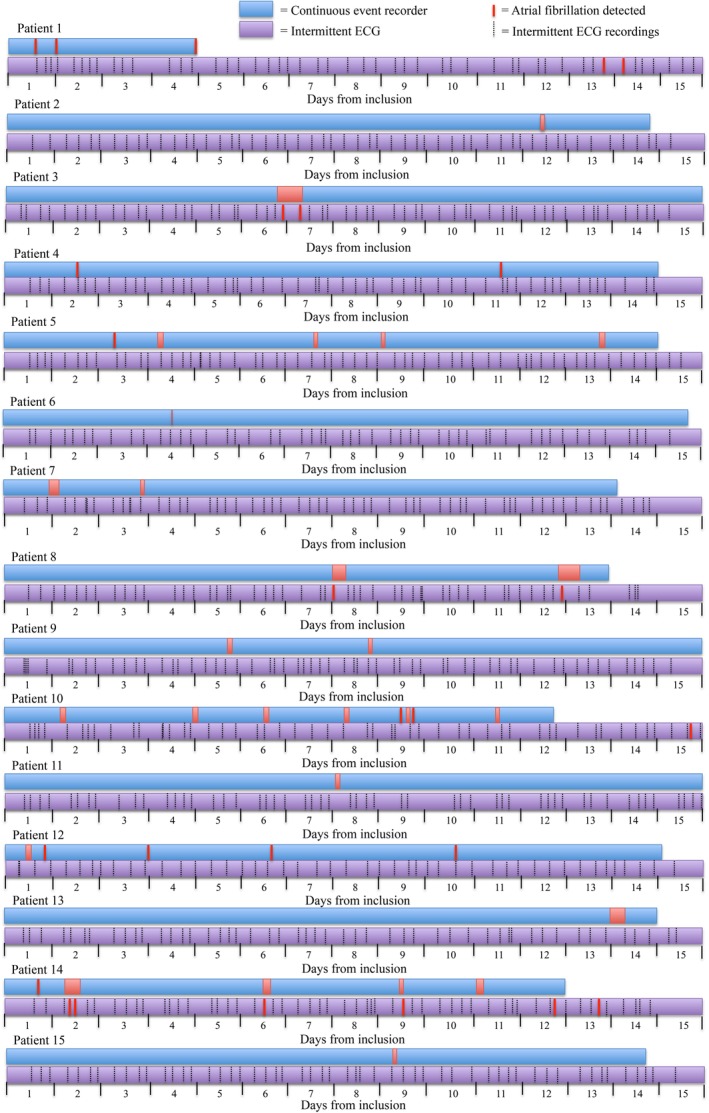
Comparison of atrial fibrillation detection per screening method

As the devices were used in parallel, arrhythmia detection was possible simultaneously. Most episodes discovered on continuous event recording were outside the detection times used for intermittent ECG recordings (Figure 2). Participants diagnosed with AF on continuous event recording had on average a total AF duration of 6 (IQR: 0‐18) hours, with an AF burden of 2 (IQR: 0‐6)%. On average, the longest individual AF episode was 32 (IQR: 4‐111) minutes.

None of the participants with AF reported palpitation symptoms on their symptom sheet during a verified AF episode. Of all participants, 6% (n = 15/269) reported symptoms in their diaries.

Individuals diagnosed with AF had lower systolic blood pressure compared to those free of AF. AF was more common in patients reporting diabetes, but was less commonly associated with hypertension, vascular disease, and previous stroke/transient ischemic attack (Table 1).

**Table 1:** Baseline characteristics at study entry

Variable	All participants (n = 269)	Participants with AF (n = 15)	Participants without AF (n = 254)	*P* value^a^
Women, n (%)	149 (55)	6 (40)	143 (56)	.077
Age (y), median (IQR)	76.5 (76.2‐77.0)	76.5 (76.0‐77.0)	76.5 (76.2‐77.0)	.681
Height (cm), median (IQR)	169 (163‐177)	173 (165‐180)	169 (163‐176)	.184
Weight (kg), median (IQR)	72 (63‐82)	72 (63‐79)	72 (63‐32)	.850
Systolic BP (mm Hg), median (IQR)	136 (127‐148)	125 (115‐135)	137 (127‐148)	.018
Diastolic BP (mm Hg), median (IQR)	82 (74‐87)	79 (70‐84)	82 (74‐88)	.139
NT‐proBNP (ng/L), median (IQR)	256 (182‐377)	257 (194‐325)	255 (181‐382)	.903
CHA_2_DS_2_‐VASc, n, median (IQR)	3 (3‐4)	3 (2‐4)	3 (3‐4)	.185
Congestive heart failure, n (%)	4 (2)	0 (0)	4 (2)	<.001
Hypertension, n (%)	138 (51)	7 (47)	131 (52)	.670
Diabetes mellitus, n (%)	33 (12)	2 (13)	31 (12)	<.001
Stroke/TIA, n (%)	23 (9)	0 (0)	23 (9)	<.001
Vascular disease, n (%)	22 (8)	1 (7)	21 (8)	<.001

Abbreviations: AF, atrial fibrillation; BP, blood pressure; CHA_2_DS_2_‐VASc, risk score for ischemic stroke; IQR, interquartile range; NT‐proBNP, N‐terminal pro b‐type natriuretic peptides levels; TIA, transient ischemic attack.

a Comparing participants with and without AF.

### 3.2 Other significant arrhythmias

In total, other significant arrhythmias were overall more commonly detected using continuous monitoring compared to intermittent recordings (Table 2).

**Table 2:** Other significant arrhythmias detected by screening method

Arrhythmia	Intermittent ECG	Continuous event recording
Second‐degree AV block Mobitz type II, n (%)	0 (0)	7 (3)
Other significant pauses, n (%)	1 (0)	10 (4)
Bradycardia, n (%)	1 (0)	1 (0)
Suspected ventricular tachycardia, n (%)	0 (0)	17 (6)

Abbreviations: AV, atrioventricular; ECG, electrocardiogram.

### 3.3 Interpretation burden per screening method

The detection algorithm for the intermittent ECG device detected all episodes manually interpreted as AF. For the continuous recorder, 73% (n = 11) of episodes manually interpreted as AF were categorized as AF by the algorithm of the device, and 27% (n = 4) were categorized as other arrhythmias. Hence, for intermittent ECGs, only episodes marked as AF needed manual interpretation, whereas for continuous ECG all categories marked as arrhythmia needed to be interpreted to detect all AF cases.

The number of ECG strips that needed manual interpretation per participant was 3 (IQR: 1‐8) for intermittent ECG compared to 55 (IQR: 40‐70) for continuous event recorder. The average time spent on analysis of ECG recordings per participant was 4.5 minutes for continuous event recording and 0.75 minutes for intermittent ECG. For continuous event recording, 4 (IQR: 0‐10)% of the ECGs were non‐interpretable compared to 0 (IQR: 0‐2)% of intermittent ECGs.

### 3.4 Compliance and patient experience

**Figure 3:**
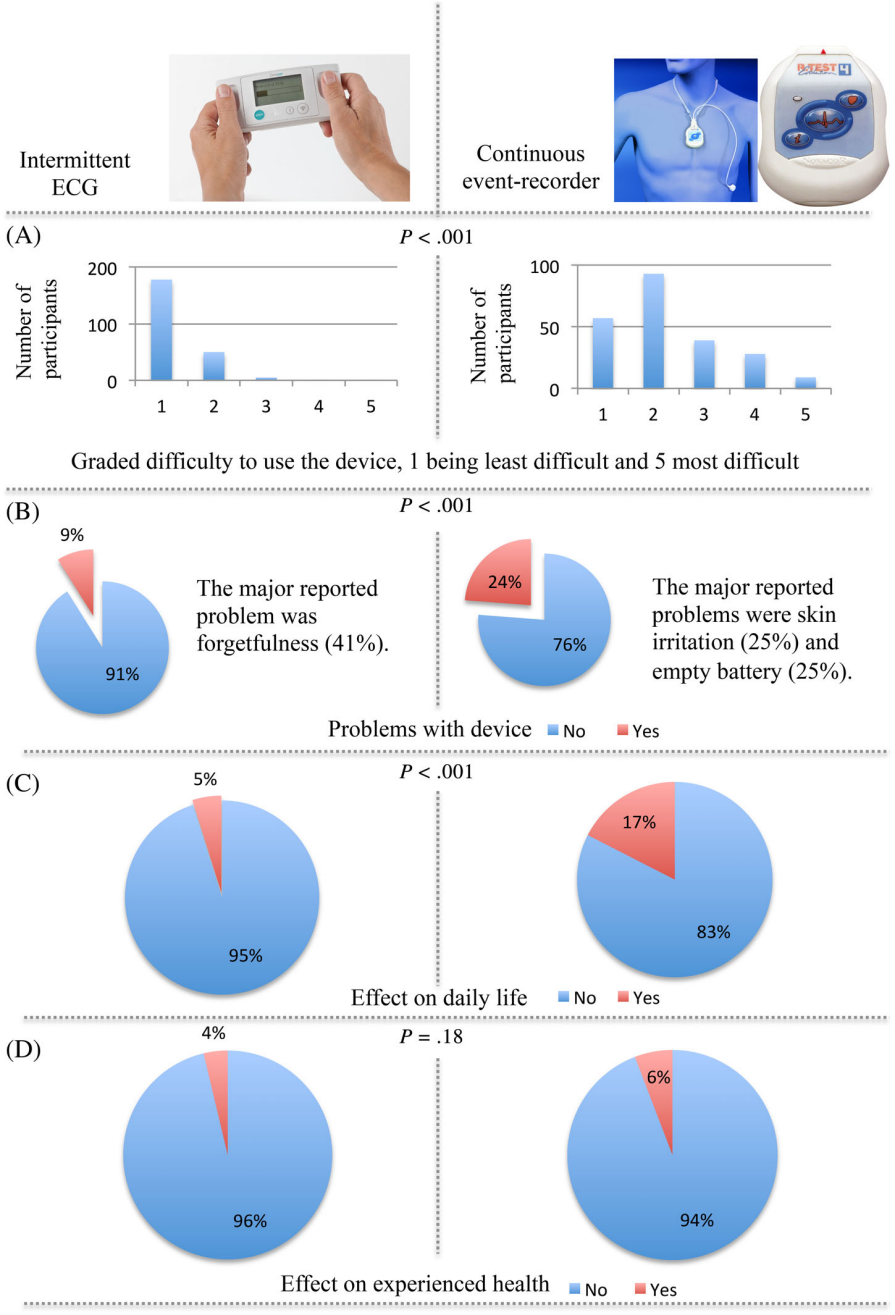
Results from forms completed by the participants regarding use of the two different screening devices. A, Grading of how difficult the device was to use. B, Experienced problems with the device. C, The electrocardiogram (ECG) registration effect on daily life. D, The ECG registration effect on how the own health is experienced

Intermittent ECG was graded as easier to tolerate compared to continuous ECG by the participants (Figure 3). Median number of intermittent ECG recordings were 55 (IQR: 40‐70) out of 56 (98% of expected). Median monitoring time for continuous event recorder was 13.1 (IQR: 11.8‐13.9) days out of 14 (94% of expected). Compliance for either device was not affected by the presence of palpitations (*P* = .559 for intermittent ECG and *P* = .804 for continuous event recording).

## 4 DISCUSSION

In this ambulatory elderly population, continuous event recording detected three times more new cases of AF compared to intermittent ECG. Both methods were well tolerated and, even in this ambulatory setting, ECG quality was good. The device‐based algorithms differed in categorization of AF events, leading to a more time‐consuming interpretation for the continuous event recording device.

Continuous event recording detected more AF cases during the first 3 days than intermittent ECG did during 2 weeks. Overall, the detection of 6% new AF is significantly higher than the outcomes of previous screening studies using intermittent ECGs (3‐4%).^11^, ^17^ From Figure 2, one can derive that not only the burden of AF is important for the detection, but also the density and duration of AF episodes. Less AF is discovered with intermittent recordings in patients with shorter intermittent episodes compared to those with single prolonged episodes. There is currently no gold standard for detection of paroxysmal AF; in this study, we have shown that it might be prudent to consider screening using continuous event recording rather than intermittent recordings, particularly in patients at high risk of stroke.

One might speculate that in order to increase the ease of use and reduce the interpretation burden for the investigator, the duration of continuous event recording could be reduced.

An important aspect of AF screening is to diagnose AF in asymptomatic patients. It might be possible that patients who experienced symptoms of palpitations were more keen to participate in our study, which might have had an impact on generalization and compliance. In a real‐world setting, compliance might be lower and the perceived differences in ease of use between the devices might lead to lower compliance for continuous monitoring.

To our knowledge, there are no prior studies comparing continuous event recording and intermittent ECG, except our smaller pilot study, where the two devices were used in parallel for 2 weeks; according to our previous results, continuous event recording detected >2.5 times more participants with AF compared to the intermittent ECG.^18^

In prior studies, both devices have shown an increased detection of AF compared to 24‐ to 48‐hour Holter monitoring. When intermittent ECG recordings, using the Zenicor device, for 10 seconds twice daily during 30 days were compared to 24‐hour continuous ECG recording, AF episodes were detected in 82% using intermittent recordings compared to 32% using continuous recording.^19^ Also, when continuous event recording using the R‐test 4 device was compared to continuous ECG, used in parallel for 48 hours, after manual interpretation of ECGs, AF was diagnosed in 4% using continuous event recording compared to 2.7% during the continuous ECG. AF was overdiagnosed using R‐test 4 due to invisible *p*‐waves.^16^

In our study, intermittent ECG was graded as more user‐friendly than continuous event recording, and previous studies have shown similar results.^20^ Also, several studies have reported disadvantages with skin irritation caused by long‐term use of external electrodes.^21^ Although problems with skin irritation and battery depletion were commonly reported for the continuous event recording device in our study, both screening devices were graded as manageable and the compliance was surprisingly high.

There is an ongoing discussion regarding the stroke risk in screening‐detected AF, as patients with screening‐detected AF may have short and rare AF episodes. In our study, screening‐detected AF was treated equally to clinically detected AF as we hypothesized that patients with AF detected during such short monitoring period are likely to have a high AF burden. In a large matched cohort study of stroke risk in incidentally detected ambulatory AF, individuals with incidentally diagnosed AF were found to have twice as high incidence of stroke compared to individuals with no AF. Anticoagulant treatment reduced stroke risk by >60% and mortality by >40% in these individuals with incidentally detected AF.^22^ Although unknown, one might hypothesize that the risk identified during this study might be representative of the risks in screening‐detected AF. The current ESC guidelines do not recommend taking AF burden or symptoms into account in stroke risk stratification, and opportunistic AF screening has a class IB recommendation.^10^

### 4.1 Limitations

The participants were all part of the STROKESTOP II study. It is possible that they were healthier than the general population, as participation in screening studies is known to be higher in healthier individuals.^23^ Compliance to the screening methods could also be increased in a highly motivated group participating in a screening study compared to the general population. All participants were elderly and most were Caucasians. In addition, only participants from STROKESTOP II with elevated NT‐proBNP levels participated in the study. This could influence the external validity of the study.

Participants with high NT‐proBNP levels are more likely to have AF, and this could lead to an increased detection, with a detection bias. As the participants are their own control, this will not affect the results of this study.

Both AF screening methods used in the study are one‐lead ECGs, making *p*‐wave analysis difficult. This could introduce a misclassification bias by underestimation of true cases. Neither of the screening devices used have 100% sensitivity for AF detection; hence, we may have underestimated the true AF prevalence.

## 5 CONCLUSION

Continuous event recording detected three times more new cases of AF compared to intermittent ECG when performed simultaneously in an ambulatory setting for 2 weeks. In our elderly population, both methods were well tolerated, although intermittent ECG was graded as more user‐friendly. The ECG quality was good for both methods, but device‐based algorithms differed in categorization of AF events, leading to a more time‐consuming interpretation for the continuous event recording device.

## CONFLICT OF INTEREST

Tove Fredriksson has received unrestricted research grants from Boehringer‐Ingelheim and Stiftelsen Hjärtat. Katrin Kemp Gudmundsdottir has received a research grant from Stiftelsen Hjärtat. Viveka Frykman has received lecture fees from MSD, Boehringer‐Ingelheim, Bayer, and Medtronic. Leif Friberg has received consultancy fees from Bayer, Boehringer‐Ingelheim, BMS/Pfizer, and Sanofi. Faris Al‐Khalili has received lecture fees from Bayer, Boehringer‐Ingelheim, and BMS/Pfizer. Johan Engdahl has received consultancy fees from Sanofi and Pfizer; lecture fees from Bayer, Boehringer‐Ingelheim, Astra Zeneca, and Medtronic; and unrestricted research grants from Pfizer and Boehringer‐Ingelheim. Emma Svennberg has received lecture fees from Bayer, Bristol‐Myers Squibb‐Pfizer, Boehringer‐ Ingelheim, and Sanofi.

## Supporting information


**Table S1.** Settings for R‐test 4 evolution.
