# Supplementary material for: Intermittent vs continuous electrocardiogram event recording for detection of atrial fibrillation—Compliance and ease of use in an ambulatory elderly population
Source: Clin Cardiol. 2020 Jan 9;43(4):355–62. doi: 10.1002/clc.23323 (PMC7144480; doi:10.1002/clc.23323)
Supplement: Supplementary file 1 — Table S1. Settings for R‐test 4 evolution. [file CLC-43-355-s001.docx]

**Supplementary table 1.** Settings for R-test 4 Evolution

| Arrhythmia | Number of episodes prioritized in storage capacity | Number of episodes displayed by software |
| --- | --- | --- |
| Ventricular tachycardia  Ventricular ectopic beats  Supraventricular tachycardia  Supraventricular ectoptic beats  Absolute pause  Relative pause  Tachycardia  Bradycardia  ST-changes  Atrial fibrillation  Marker (activated by the patient) | 4  4  10  5  10  4  8  3  0  42  8 | 10  10  10  10  10  10  10  10  0  42  10 |
